# Exogenous Lactogenic Signaling Stimulates Beta Cell Replication In Vivo and In Vitro

**DOI:** 10.3390/biom12020215

**Published:** 2022-01-26

**Authors:** Katelyn Millette, Keith Rodriguez, Xia Sheng, Stacey D. Finley, Senta Georgia

**Affiliations:** 1 Department of Stem Cell Biology and Regenerative Medicine, Keck School of Medicine, University of Southern California, Los Angeles, CA 90033, USA; millette@usc.edu; 2 Center for Endocrinology, Diabetes and Metabolism, Department of Pediatrics, Children’s Hospital Los Angeles, Los Angeles, CA 90027, USA; keithrodriguez22@gmail.com (K.R.); ephoton17@yahoo.com (X.S.); 3 Mork Family Department of Chemical Engineering and Materials Science, University of Southern California, Los Angeles, CA 90089, USA; sfinley@usc.edu; 4 Departments of Biomedical Engineering and Quantitative and Computational Biology, University of Southern California, Los Angeles, CA 90089, USA

**Keywords:** beta cells, diabetes, regeneration, replication, gestational diabetes, pregnancy, insulin

## Abstract

As patients recently diagnosed with T1D and patients with T2D have residual beta cell mass, there is considerable effort in beta cell biology to understand the mechanisms that drive beta cell regeneration as a potential cellular therapy for expanding patients’ residual beta cell population. Both mouse and human studies have established that beta cell mass expansion occurs rapidly during pregnancy. To investigate the mechanisms of beta cell mass expansion during pregnancy, we developed a novel in vivo and in vitro models of pseudopregnancy. Our models demonstrate that pseudopregnancy promotes beta cell mass expansion in parous mice, and this expansion is driven by beta cell proliferation rather than hypertrophy. Importantly, estrogen, progesterone, and placental lactogen induce STAT5A signaling in the pseudopregnancy model, demonstrating that this model successfully recapitulates pregnancy-induced beta cell replication. We then created an in vitro model of pseudopregnancy and found that the combination of estrogen and placental lactogen induced beta cell replication in human islets and rat insulinoma cells. Therefore, beta cells both in vitro and in vivo increase proliferation when subjected to the pseudopregnancy cocktail compared to groups treated with estradiol or placental lactogen alone. The pseudopregnancy models described here may help inform novel methods of inducing beta cell replication in patients with diabetes.

## 1. Introduction

During pregnancy, the expectant mother is highly insulin resistant. This insulin resistance results from many factors, including adipose tissue, placental growth, and pregnancy hormones [1]. To respond to the increased metabolic demands of pregnancy, female beta cells must undergo expansion and involution after parturition. Throughout multiple pregnancies, female beta cells must undergo multiple cycles of expansion and involution. While the increase in maternal blood glucose aids fetal growth, failure to sufficiently expand functional beta cell mass can result in gestational diabetes (GD), thereby complicating the pregnancy and delivery. Women have a 2–9% chance of being diagnosed with GD during their first pregnancy; however, the risk changes during subsequent pregnancies [2]. If the mother was not diagnosed with gestational diabetes during her first pregnancy, the risk of GD drops significantly with her second pregnancy. However, women previously diagnosed with GD have up to 63% (depending on compounding risk factors) risk of GD reoccurrence [3,4,5]. These clinical data suggest that beta cells that successfully adapted to insulin demands during a previous pregnancy can do so again during additional pregnancies. Therefore, it is imperative that we understand the mechanisms that regulate beta cell expansion during pregnancy. Exploiting these mechanisms could have a significant impact on developing therapies to expand beta cells in vivo for patients with all forms of diabetes.

The mechanism of human beta cell expansion during pregnancy is debated in the field, and research is limited to in vitro islet culture and epidemiological studies on mothers with SNPs in the prolactin receptor (PRLR) [6,7]. Human in vitro islet studies demonstrated that beta cells replicate in response to prolactin (PRL), and the epidemiological study reported that PRLR SNPS are associated with gestational diabetes. Mouse models have concluded that increases in maternal beta cell proliferation are mediated by lactogenic signaling via PRL and placental lactogen (PL) through PRLR. Constitutively active PRLR in mouse beta cells stimulates beta cell replication and increases beta cell mass; conversely, insufficient lactogenic signaling by loss of PRLR restricts the capacity of beta cells to expand during pregnancy resulting in gestational diabetes [8]. Mechanistically, PRL binds to PRLR and activates STAT5 signaling; this increases the expression of factors important for beta cell proliferation, function, and survival [9,10]. While there is in vitro evidence that PRL levels are responsible for the increase in insulin secretion observed during human pregnancy, the mechanisms behind these findings remain largely unstudied [10,11].

While the models of PRL overexpression and PRLR activation have been informative, their conclusions are confounded by multiple factors in the endogenous pregnancy milieu [8,12,13,14,15]. Litter size dictates exposure to PL expression during pregnancy, making it difficult to titrate PL concentrations in vivo to understand how PL activates PLR signaling. Therefore, we modified a well-established pseudopregnancy protocol to specifically study the effects of PL on beta cell regeneration. Studies from the mammary gland induce pseudopregnancy by implanting tablets that contain estrogen and progesterone (referred to as estrogens E, going forward); this simulates high levels of estrogen and progesterone during pregnancy and stimulates mammary gland proliferation and remodeling [16,17] Because it has been established that PRL activation of PRLR stimulates beta cell proliferation and estrogen increases beta cell survival, we adapted the pseudopregnancy protocol to include both estrogen exposure and PRLR stimulation in a temporal sequence similar to what has been documented in normal rodent pregnancy [18]. We used exogenous chemical hormones to induce pseudopregnancy to standardize estrogen and PL exposure, stimulate the key hormonal pathways of pregnancy that activate beta cell expansion, and allow the accurate comparison of treatment groups in vivo [19] We developed a model of the hormonal pregnancy milieu by administering a cocktail of E and PL. We subjected rat INS1 cells and dispersed human islets from healthy female donors to the pseudopregnancy cocktail in vitro. Here we show that these models can be used to answer mechanistic questions about beta cell replication and the beta cell proliferative response to pregnancy.

## 2. Materials and Methods

### 2.1. Mice

All animal studies were approved and completed in accordance with CHLA Institutional Animal Care and Usage Committee. C57BL/6 female mice (Jackson Laboratry, Bar Harbor, ME, USA) were all 16 weeks of age at the beginning of experiments. Parous mice were all retired breeders after two or more litters, and nulliparous had never been pregnant.

### 2.2. In Vivo Pseudopregnancy Model: Pellet Implantation and PL Injections

Mice parous and nulliparous mice were randomly assigned to treatment groups on Day 0. For the females who received estrogen treatments, 17β-estrogen (0.5 mg) and progesterone (10 mg) slow-release tablets (Innovative Research of America, Sarasota, FL, USA) were surgically inserted subcutaneously between the shoulder blades of age-matched parous and nulliparous female mice on Day 0. For the females that received placental lactogen supplementation, mice received intraperitoneal injections of 0.5 ug of placental lactogen (Sigma, St. Louis, MO, USA) 2× daily for 4 days, beginning on Day 7. Injections are given once in AM and once in PM, 10–12 h apart. Both nulliparous and parous control animals received no estrogen or placental lactogen treatments.

### 2.3. Histology

Histology was performed as previously described [20]. Briefly, freshly isolated pancreata were fixed in 4% PFA, embedded in paraffin, and sectioned at 5 μm onto charged slides. Slides were rehydrated using toluene and decreasing concentrations of ethanol. We used a microwave and citrate buffer method of antigen unmasking, TBS + 0.4% Triton for permeabilization for 30 min, and blocked non-specific staining using TBS + 2%BSA + 0.2% Tween for 1 h. All primary and secondary antibodies were diluted in blocking buffer. Slides were incubated with primary antibody overnight at 4 °C, and all slides were incubated with secondary antibodies for 30 min at room temperature.

Antibodies used: Insulin (DAKO, #A0564, Santa Clara, CA, USA), PRLR (Bioss, # bs-6445R, Woburn, MA, USA), E-cadherin (Cell Signaling Technology, #24E10, Danvers, MA, USA), STAT5A (Abcam, #ab7969, Cambridge, UK).

### 2.4. Histology Quantification

Beta cell mass: Tile scanned 5× images were stitched and analyzed in FIJI/ImageJ for analysis. Images were cropped using the freehand selections tool to exclude lymph nodes and blood vessels. We then set the image to binary and dilate settings before measuring the area for the DAPI and Insulin channels separately. We then divided the insulin channel area by the DAPI channel area and multiplied it by 100 to get the beta cell mass percentage. *n* = 3–6.

Beta cell area: Islets stained for E-cadherin and Insulin were imaged at 20× and analyzed in FIJI/ImageJ. We used the freehand selections tool to follow the E-cadherin outline of individual cells in an islet and used the fill tool to fill in the cells. We then measured the area of the filled-in cells using the quantitative software package in ImageJ.

### 2.5. Cell Culture

The INS1E cell line was maintained in RPMI 1640 supplemented with glutamine, 10% FBS, 1% sodium pyruvate, 1% penicillin/streptomycin, and 0.1% beta-mercaptoethanol. Cells were split at 70% confluency.

Islet donor profiles were screened to select female donors with recorded documentation of childbearing. Human islets (Prodo labs) were washed twice upon arrival and cultured in RPMI 1640 supplemented with 10% FBS, 1% Glutamax, 1% penicillin/streptomycin, and 1 mM nicotinamide. Islets were dispersed using TrypLE between 24–48 h after arrival for in vitro pseudopregnancy assay.

#### In Vitro Pseudopregnancy Proliferation Assay

INS1E cells were seeded at 20,000 cells/cm^2^. Dispersed human islets were seeded for an optimal 40–50% starting confluency. The day after seeding, cells were rinsed with PBS, and media was refreshed (D0). On Day 1, we added 10^−8^ M 17β-estrogen (Steraloids). On Day 2, the media was changed and supplemented with 10^−8^ M estrogen. On days 3, 4, and 5, fresh media supplement with either estrogen (10^−8^ M), placental lactogen (500 ng/mL), or both. On day 5, 25 μM of EdU for human islets and 10 μM EdU for INS1E cells (Click-iT EdU Kit, Thermofisher Cat. # C10646, Waltham, USA) was added to the media and incubated for 4 h before fixation. Click-iT reaction was performed as per the manufacturer’s protocol. Dispersed human islet cells were costained with insulin to identify beta cells. We included an untreated control well, as well as a positive control well treated with CHIR99021 (Tocris, Bristol, UK), to stimulate proliferation [21]) on days 3–5. At least 500 cells from 4 different quadrants of each plate were counted and quantified for EdU+ proliferation (total 2000 cells per well). Experiments were repeated as *n* = 3 biological replicates; each experiment ran with 2 technical replicates.

### 2.6. Statistical Analysis

All statistical analysis was performed using GraphPad Prism 8. For experiments where samples had equal variance, a one-way ANOVA was used, followed by Tukey’s Multiple Comparison’s test to compare individual groups with each other. We used the Kruskal–Wallis non-parametric test for experiments where samples had unequal variance.

## 3. Results

### 3.1. Pseudopregnancy Promotes Beta Cell Proliferation in Multiparous Mice In-Vivo

Estrogen (E), progesterone, and placental lactogen (PL) rise and fall throughout pregnancy to stimulate physiologic support for the mother and fetus. To better understand the synergistic effect of these hormones on beta cell adaptation during pregnancy, we adapted a well-studied model of pseudopregnancy from the mammary gland biology that delivers hormones at levels comparable to those measured during mouse pregnancy and successfully mimicked the effects of pregnancy, including injections of PL that have been shown to induce beta cell proliferation in vivo [17,18,22]. Slow-release estrogen tablets were surgically implanted subcutaneously on day 0, and from days 7–10, mice received two injections of PL per day. Mice were euthanized on day 11 (Table 1). We used eight treatment groups to determine if there were synergistic roles E and PL during the beta cell mass expansion of both parous and nulliparous mice.

**Figure 1:**
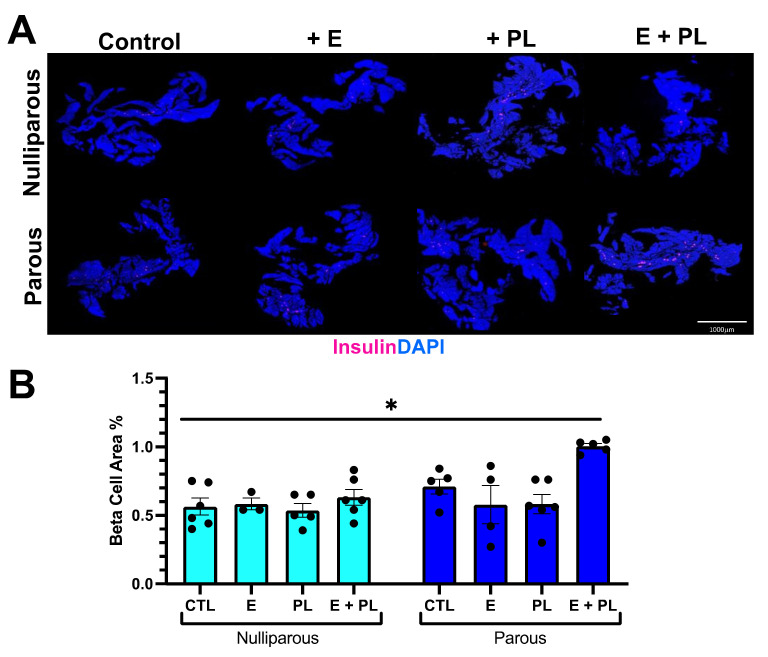
Pseudopregnancy promotes beta cell mass expansion in parous mice. (**A**) Representative pancreata stained with insulin (magenta) and DAPI (blue) from each treatment group, 5× tile images stitched using ImageJ. (**B**) Quantification of beta cell mass (insulin/DAPI ratio); *n* = 3–6, * *p* = 0.02 using Kruskal-Wallis non-parametric test.

**Figure 2:**
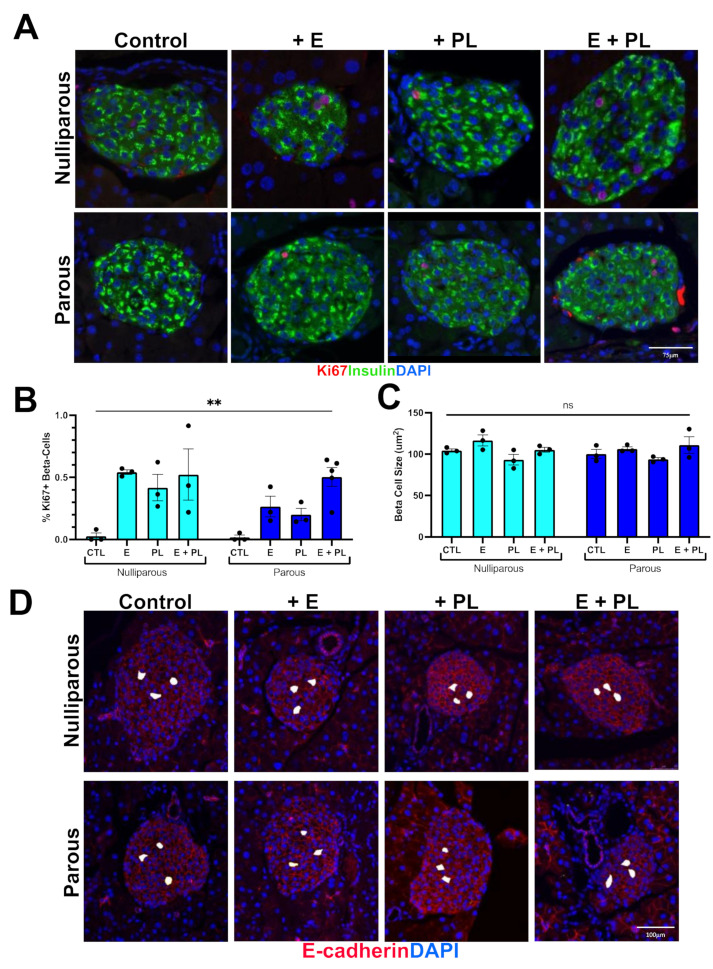
Pregnancy hormones induce beta cell proliferation but not hypertrophy in mice. (**A**) Immunohistochemical staining for the replication marker Ki67 (red) and insulin (green) at 20× magnification. (**B**) Statistical analysis of Ki67+ beta cells; *n* = 3–5, ** *p* = 0.01 using Kruskal–Wallis non-parametric test. (**C**) No change (ns) in beta cell area was found in any group; ANOVA *p* = 0.09, *n* = 3. (**D**) Islets stained with E-cadherin; white blocked cells represent beta cell area quantification method (see methods section).

To assess whether our pseudopregnancy model stimulated adaptive beta cell expansion, we quantified beta cell area from both parous and nulliparous female cohorts. Controls from nulliparous and parous mice had no significant difference in the beta cell area. The beta cell area of nulliparous mice treated with the cocktail was not statistically different from untreated control mice. Parous mice treated with the pseudopregnancy cocktail (E + PL), however, had almost a twofold increase in the beta cell area compared to treated nulliparous mice (Figure 1). To assess whether the increase in beta cell area was due to proliferation or beta cell hypertrophy, we stained histological sections with either insulin and the replication marker Ki67 or insulin and E-cadherin (Figure 2). Histological analysis confirmed that the increase in beta cell area was due to the proliferation of beta cells. Parous pseudopregnant mice had a 163.7% increase in proliferation compared to the nulliparous control. We also saw increases in proliferation with the addition of E and PL in both nulliparous and parous females; however, these increases in proliferation do not translate into increased beta cell mass except in the parous pseudopregnant cohort. These proliferation increases may suggest that either the replicative cells are not maintained, or there is incomplete replication. To assess if the replicating cells were eliminated, we quantified the expression of cleaved caspase 3, an early marker of cell death, in beta cells. Cleaved caspase 3 expression was very infrequent and was not statistically different in any of the cohorts (data not shown). Beta cell hypertrophy was quantified by measuring cell area as marked by E-cadherin; examples of beta cell area analysis can be found in Figure 2D. There was no significant difference in beta cell hypertrophy between pseudopregnant groups (Figure 2). Parous mice who did not have an induction of pseudopregnancy had statistically significant smaller beta cells than all other cohorts.

**Figure 3:**
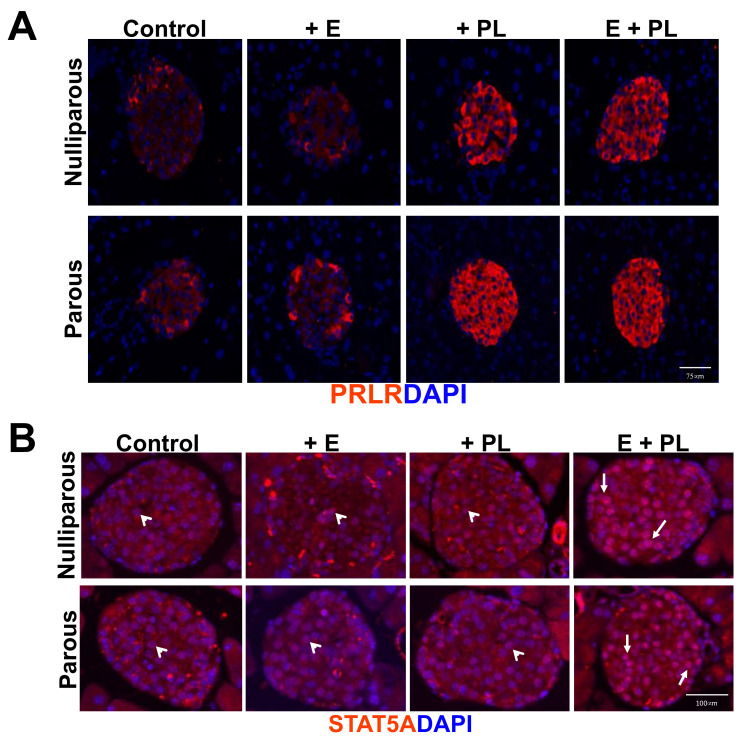
E + PL induce STAT5 signaling in the pseudopregnancy model. (**A**) Immunohistochemical staining for PRLR in nulliparous (top) and parous (bottom) mouse islets. (**B**) STAT5 expression in nulliparous (top) and parous (bottom) mouse islets. Open arrowheads show minimal accumulation, while white arrows show maximal accumulation.

Many studies have shown that adaptive beta cell proliferation is mediated by PRL stimulating PRLR. When PRL binds to PRLR, the downstream target genes important for beta cell proliferation, function, and survival are upregulated. This is mediated through STAT5A activation and translocation into the nucleus [9,20]. To understand if the increase in beta cell proliferation was mediated through this pathway in our pseudopregnancy model, we performed immunofluorescent staining for both PRLR and STAT5. We found that PRLR expression increases with exposure to PL regardless of previous pregnancies (Figure 3). These results indicate that beta cells respond to PL by increasing PRLR receptor expression. Next, we investigated whether STAT5 signaling was activated. We found higher levels of nuclear STAT5 accumulation in the mice exposed to both estradiol and placental lactogen, regardless of previous pregnancy (Figure 3).

### 3.2. Pseudopregnancy Induces Beta Cell Replication In Vitro

**Figure 4:**
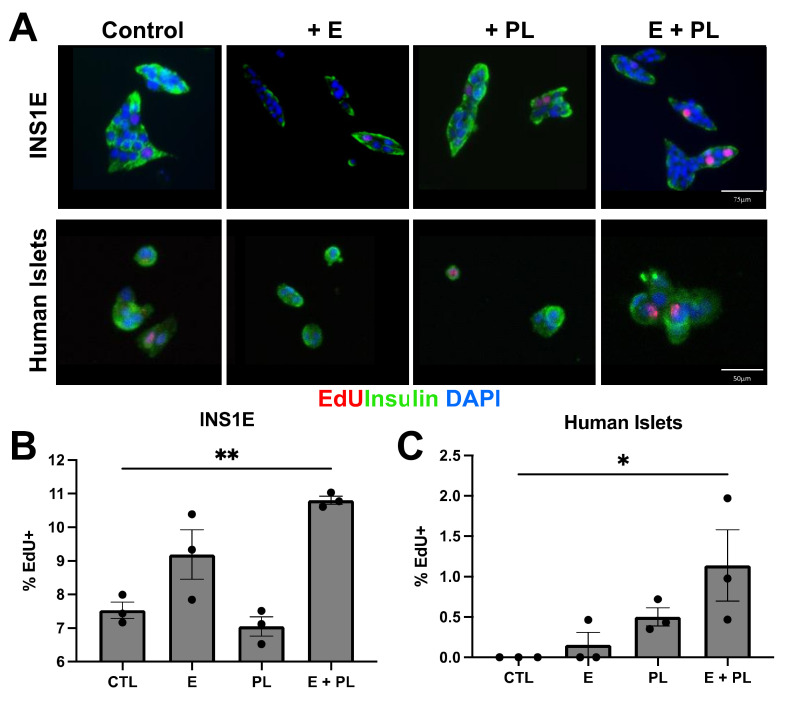
In-Vitro pseudopregnancy model promotes beta cell replication in human islets and INS1E cells. (**A**) Representative immunocytochemistry showing EdU incorporation (red) in beta cells (green) from the INS1E cell line (top row), and female human islets (bottom row) after treatment with E and PL. (**B**) Quantification of the proliferation of INS1E cells under the pseudopregnancy assay. INS1E control group and E + PL group (** *p* < 0.01) as well as between PL and E + PL (*p* < 0.05) showed significant differences, *n* = 3. (**C**) EdU incorporation in female human islets treated with E and PL were statistically different from untreated control cells (* *p* < 0.05 by ANOVA); *n* = 3.

To determine whether our model of in-vitro pseudopregnancy was helpful in other species and in male islets, we tested the cocktail on rat insulinoma cell line INS1E. We found the E + PL treatment results in a 1.43-fold increase in EdU incorporation relative to the control, suggesting that male beta cells may also positively respond to these hormonal cues (Figure 4). Our in vitro pseudopregnancy model induces beta cell replication reliably regardless of cell line gender.

While there is evidence in mouse models that placental lactogen signaling plays a critical role in regulating beta cell proliferation during pregnancy in mice, there have not been practical ways to study whether this occurs during human pregnancy. Brelje and colleagues reported an increase in insulin secretion and beta cell replication in mouse, rat, and human islets in vitro in the presence of placental lactogen and placental lactogen receptors [11]. We sought to mimic the beta cells’ response to pregnancy in vitro by treating human islets from non-diabetic female donors with a pseudopregnancy cocktail containing E (10^−8^ M) and PL (500 ng/mL). Because the parous females responded to the pseudopregnancy cocktail in vivo (Figure 1, Figure 2 and Figure 3), we chose female donors who had documentation in their donor records of having children. Islets were dispersed, plated, and treated with E for four days; PL as added in days three and four. Cells were treated with EdU, a marker for cellular proliferation, for 4 h before fixation on day five. The description of experimental groups can be found in Supplementary Figure S1. We performed this experiment with three different donor samples and technical replicates. Our data suggest that while PL alone promotes moderate EdU incorporation, the combination of E and PL induces a 127% increase in dispersed human beta cell replication as marked by EdU incorporation (Figure 4).

## 4. Discussion

Here we present a novel model of lactogenic signaling that we used to study the synergistic relationship of estrogen and placental lactogen in stimulating beta cell proliferation. While previous studies have relied on knockout receptor and transgenic overexpression models, these are the first models that allow researchers to investigate phenotypic changes based on direct signaling through the hormones themselves.

Fully mature beta cells rarely replicate unless presented with physiological stress. This model sought to replicate the hormonal milieu of pregnancy without the confounding stress factors, such as insulin resistance or parity, to study the hormonal mechanisms that mediate beta cell proliferation. We developed a novel model of pseudopregnancy that used exogenous hormones to activate key hormonal signaling pathways known to mediate beta cell replication in vivo. By administering exogenous hormones, we were able to standardize hormone exposure in a way that is not possible during normal pregnancy; the facilitated reproducible results and isolate specific pathways can drive beta cell adaptation. In our model, we measured increases in beta cell proliferation in pseudopregnant nulliparous and parous mice, but beta cell area only increased in pseudopregnant parous mice. The increase in beta cell mass in parous mice exposed to E + PL is due to the proliferation of existing beta cells rather than the expansion of beta cell size. This opens the possibility that parity confers an adaptive advantage on beta cells. Further work is underway to identify the pathways that allow parous beta cells to adapt and/or restrict the capacity of nulliparous beta cells to adapt.

Because only parous animals showed a response to the pseudopregnancy cocktail, we subjected human beta cells from parous female donors to the pseudopregnancy cocktail. Human beta cells showed a trend towards increased proliferation when subjected to the pseudopregnancy cocktail. These results could have been confounded by age or underlying health conditions that compromised or restricted beta cell function prior to death. We could not confirm if female donors were nulliparous and, thus, were unable to assess the appropriate controls for comparison. Taken together, our in vivo and in vitro results suggest that a previous pregnancy leads to enhanced capacity for beta cell proliferation in subsequent pregnancies. It is possible that pregnancy results in epigenetic remodeling that facilitates proliferation in subsequent pregnancies; this concept has been illustrated in the mammary gland and warrants further investigation.

The increased expression of PRLR, nuclear STAT5 accumulation, beta cell replication, and beta cell mass expansion are consistent with beta cell responses to pregnancy and suggest that our in vivo pseudopregnancy cocktail induces replication by stimulating the pathways that increase beta cell mass during pregnancy in mice. Both nulliparous and parous beta cells accumulated STAT5A in the nucleus, but only parous animals responded by increasing beta cell mass. This change in responsiveness may depend on epigenetic remodeling during pregnancy that is maintained post-pregnancy and should be further investigated. While we were able to show STAT5A accumulation in the nucleus, treating animals or islets with inhibitors against upstream kinases in the PRLR pathway, such as p-JUN or ERK, would provide more conclusive evidence that beta cell proliferation is dependent on STAT5A. However, earlier studies have shown that lactogens promote beta cell survival and proliferation through STAT5A and support our conclusions [9,23].

These findings are significant since understanding endogenous regeneration mechanisms will be important in understanding how to induce beta cell replication in vivo. Functional tests, such as intraperitoneal glucose tolerance test and insulin tolerance test, are needed to evaluate whether this increase in beta cell mass translates into differences in glucose sensitivity. Our group has used this work to develop a mechanical computational model to describe prolactin-mediated JAK-STAT signaling in pancreatic beta cells and reveal possible strategies to modulate STAT5 signaling [24]. Using such a model trained with experimental data could lead to the development of novel therapeutic strategies to expand beta cell mass.

## Figures and Tables

**Table 1:** Treatment Groups. Experimental design and nomenclature of in vivo treatment groups. Nulliparous and parous female mice were subdivided into 8 different groups comprised and received either a slow-release estrogen tablet implantation, placental lactogen injections, or both. Control animals underwent sham surgery and/or PBS injections. Nomenclature describes the treatments and treatment days used in in vivo pseudopregnancy model and subsequent figures.

Nulliparous	Parous
Nulliparous + Estrogen (D0-D11)	Parous + Estrogen (D0-D11)
Nulliparous + Placental Lactogen (D7-D11)	Parous + Placental Lactogen (D7-D11)
Nulliparous + Estrogen (D0-D11) + Placental lactogen (D-D11)	Parous + Estrogen (D0-D11) + Placental lactogen (D7-D11)

## Supplementary Materials

The following are available online at https://www.mdpi.com/article/10.3390/biom12020215/s1, Figure S1: Experimental design and nomenclature for in vitro treatment groups.
