# Supplementary material for: Exogenous Lactogenic Signaling Stimulates Beta Cell Replication In Vivo and In Vitro
Source: Biomolecules. 2022 Jan 26;12(2):215. doi: 10.3390/biom12020215 (PMC8961548; doi:10.3390/biom12020215)
Supplement: Supplementary file 1 [file biomolecules-12-00215-s001.zip › biomolecules-1517357-supplementary.pdf]

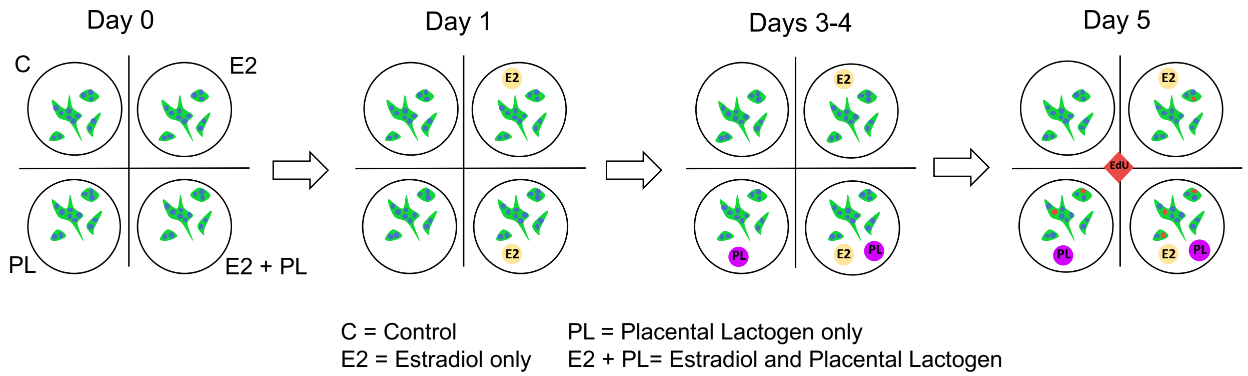

Supplementary Figure S1. Experimental design and nomenclature for in vitro treatment groups. Media was refreshed daily.
